# Explaining the Association between Driver’s Age and the Risk of Causing a Road Crash through Mediation Analysis

**DOI:** 10.3390/ijerph17239041

**Published:** 2020-12-04

**Authors:** Karoline Gomes-Franco, Mario Rivera-Izquierdo, Luis Miguel Martín-delosReyes, Eladio Jiménez-Mejías, Virginia Martínez-Ruiz

**Affiliations:** 1 Department of Preventive Medicine and Public Health, University of Granada, 18016 Granada, Spain; karolinefranco@yahoo.com.br (K.G.-F.); luismiguelmr@ugr.es (L.M.M.-d.); eladiojimenez@ugr.es (E.J.-M.); virmruiz@ugr.es (V.M.-R.); 2 Service of Preventive Medicine and Public Health, Hospital Universitario San Cecilio, 18016 Granada, Spain; 3 Biomedical Network Research Centers of Epidemiology and Public Health (CIBERESP), ISCIII, 28029 Madrid, Spain

**Keywords:** younger drivers, older drivers, road crash, mediation analysis, risk factors

## Abstract

It has been widely reported that younger and older drivers have an excess risk of causing a road crash. Two casual hypotheses may coexist: the riskier driving behaviors and age-related mechanisms in extreme age groups (direct path) and the different environmental and vehicle circumstances (indirect path). Our aim was to quantify, through a mediation analysis, the percentage contribution of both paths. A case-control study was designed from the Spanish Register of Road Crashes with victims from 2014 to 2017. Assuming a quasi-induced exposure approach, controls were non-responsible drivers involved in clean collisions between two or more vehicles (*n* = 52,131). Responsible drivers for these collisions plus drivers involved in single crashes constituted the case group (*n* = 82,071). A logit model in which the outcome was the log (odds) of causing a road crash and the exposure was age groups was adjusted for driver, vehicle and environmental factors. The highest crash risk was observed in extreme age groups, compared to the 35–44 year old age group: the youngest (18–24 years old, odds ratio = 2.14, 95% confidence interval: 2.06–2.24) and the oldest drivers (>74 years old, odds ratio = 3.30, 95% confidence interval: 3.04–2.58). The mediation analysis identified the direct path as the main explanatory mechanism for these increases: 89% in the youngest and 93% in the oldest drivers. These data support the hypothesis that the excess crash risk observed for younger and older drivers is mainly related to their higher frequency of risky driving behaviors and age-related loss of capabilities. Preventive strategies in extreme-aged drivers should focus on decreasing these behaviors.

## 1. Introduction

### 1.1. Literature Review

Several studies have reported increased road crash rates for both younger and older drivers (under 25 and over 65 years old, respectively), compared to middle-aged drivers. The reasons given for both increases have been widely explored [1,2]. For younger drivers, these are mainly related to inexperience and risk-taking behaviors (driving under the influence of alcohol and other drugs, speeding, etc.) [1,3,4,5,6,7,8,9]. This excess of riskier behaviors and higher traffic incident rates has also been suggested for younger cyclists [10]. As for older drivers, their excess risk has usually been related to their reduced ability to cope with the inherent complexities of driving—a situation causally associated with three age-related factors: (a) the physiological loss of capabilities and age-related fragility [2], (b) the pathological loss of capabilities derived from age-related illnesses such as dementia and other mental pathologies, visual and hearing defects, etc. [11,12], and (c) the frequency of driving under the influence of drugs that affect the driver’s abilities [13]—a frequency reported to be higher in older drivers [14].

However, some researchers have proposed several alternative hypotheses to partially or even completely explain these age-related risk increases They all share a common background: a significant part of the risk posed by each category of driver is related to the amount and type of exposure to the risk [15,16,17], which is linked to an intrinsically high crash risk, regardless of the driver’s characteristics. Therefore, to compare the crash risks yielded by, for example, the age categories of drivers, it is first mandatory to adjust this risk by the amount and type of exposure yielded by each driver’s age category. A common example to illustrate the failure of the above requirement is the low mileage bias [17,18]: although crash rates for different driver subgroups were estimated for a fixed amount of exposure (measured as time spent on the road, or more frequently, as distance traveled) [16], this is not a fair comparison, as distances traveled on highways or motorways, and long journeys in general, are associated with lower crash risk than distances traveled in urban areas and short journeys.

It is well-known that older drivers, unlike younger ones, accumulate their travel distances in short (low-mileage) trips mostly in urban areas, where the risk of being involved in an accident is intrinsically higher [19]. Another example is the type of vehicle driven: if, for example, extreme age groups of drivers use older vehicles more frequently (intrinsically associated with a higher crash risk) compared to middle-aged drivers, a biased comparison between age-related crash risks would result.

### 1.2. Assumptions

**Figure 1:**
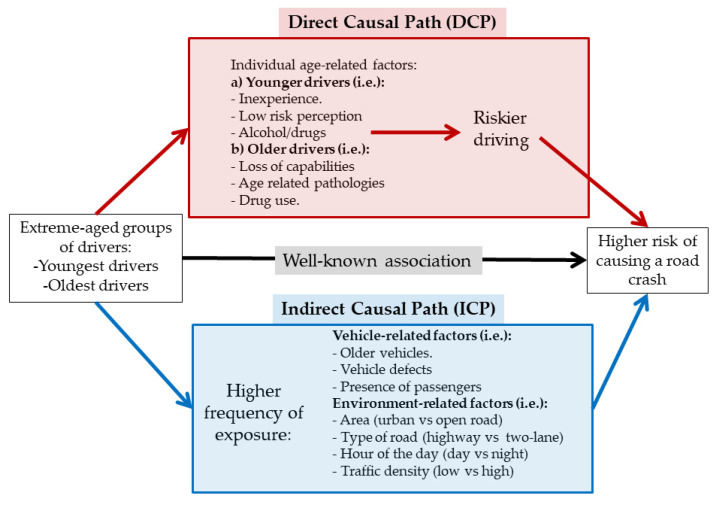
The two possible casual paths that could explain the association between drivers’ age and the risk of causing a road crash. Directed acyclic graph (DAG).

A general formulation of all previous causal associations regarding age-related increases in crash risk implies the a priori assumption of two causal paths linking age with a high risk of causing a road crash (Figure 1):

- A direct causal path (DCP). In this path, the driver’s age is associated with the risk of a crash regardless of the amount and type of exposure (the road, the time of the day, the type of vehicle driven, etc.). The reasons for this DCP would be those described in the first paragraph of this introduction for both younger and older drivers. Ultimately, all of these circumstances lead to a loss of optimal driving capabilities or to riskier driving behavior;
- An indirect causal path (ICP). In this path, the driver’s age is associated with an increased crash risk because it is causally associated with a riskier driving environment or a riskier vehicle: for example, younger drivers tend to drive more frequently at night, while aged drivers tend to drive more frequently on urban roads.

In order to establish intervention priorities for (theoretically) high-risk groups consisting of younger and older drivers, it seems very relevant to know which part of this high crash risk is related to each of the two causal paths described above. Therefore, depending on the possible results, preventive strategies could focus either on changing driving behaviors of extreme-aged drivers (increasing information, consciousness, sanitary advice, etc.) or identifying their loss of capabilities, if the direct path prevails, or in changing the driving environment and vehicle conditions of these drives if the indirect path predominates.

### 1.3. Hypotheses and Objectives

The research question of this work, therefore, is what percentage of the excess of risk in extreme-aged groups corresponds to each of the two casual paths. Our hypothesis was that non-related vehicle and environmental factors (that is, the DCP) are the main explanatory cause of excess risk in extreme-aged drivers. To our knowledge, no previous studies aimed at this purpose, although investigating this would be relatively easy by applying an analytic approach known as mediation analysis. The novelty of this study lies in the use of this method in a large sample of drivers in Spain. Therefore, the objectives of the present study are:

- To confirm the excess risk of younger and older drivers of causing a crash compared to middle-aged drivers;
- If this excess risk is confirmed, the second aim is to quantify which part of this higher risk is related to a DCP and which part depends on an ICP, by applying a mediation analysis based on a decomposition method.

## 2. Materials and Methods

### 2.1. Data Used in the Study

We designed a retrospective case-control study using data from the Spanish Register of Road Crashes from the Spanish Traffic Directorate for the years 2014 to 2017. It is a nationwide police-based register of all road crashes with victims. The characteristics of this register have been described elsewhere [20,21]. Three of the variables included are the type of crash, the type of vehicle and the commission of infractions or driving errors immediately prior to the crash by any driver involved. Taking into account this information, we defined our original study sample as that comprised by the 134,202 drivers of four-wheeled vehicles (cars, vans and all-terrain vehicles) involved in road crashes ascribed to any of the following three subgroups:

- Subgroup 1. Drivers involved in single crashes in which only one moving vehicle was involved (*n* = 31,290 drivers);
- Subgroup 2. Offender drivers (drivers who were at fault for the crash), involved in clean collisions (i.e., collisions between two or more moving vehicles, including frontal, front-lateral, lateral, rear or multiple collisions) in which only one of the drivers involved committed a traffic infraction or error immediately prior to the crash) (*n* = 50,781 drivers involved in as many clean collisions);
- Subgroup 3. Non-offender drivers (drivers who were not at fault for the crash) involved in the 50,781 clean collisions described above (*n* = 52,131 drivers).

As several drivers presented missing values in some of the variables evaluated, the sample analyzed in this study finally consisted of 118,364 drivers with complete records for all variables.

We assumed that most drivers in subgroups 1 and 2 were responsible for the crash in which they were involved; therefore, they comprised the case group. As can be noticed in the three subgroups, only single or clean collisions were considered in the study. Therefore, incidents in which there were two or more drivers who committed an infraction were not included in the case-control study. On the other hand, most drivers included in the subgroup 3 were innocent and could be considered a representative sample of moving drivers on the road; therefore, they constituted the control group. This quasi-induced approach, recently validated as an appropriate way to select the reference group in traffic databases [22], has been widely used in previous studies aimed at comparing the risk of road crashes across subgroups of drivers [23,24,25]. An advantage of the quasi-induce exposure method is that it indirectly allows the strength of the association between age and the risk of causing a traffic accident to be adjusted according to the amount of exposure to driving, without the need to restore to direct estimation (e.g., using time measures or distance traveled by each driver).

### 2.2. Main Variables Considered

For each driver/vehicle/environment/crash we considered the following variables, obtained from the information provided in the register:

- Driver variables: Age (<25, 25–34, 35–44ref, 45–54, 55–64, 65–74, >74) sex;
- Vehicle variables: Type (cars, vans, all-terrain vehicles), years since the vehicle was registered (0–4, 5–9, 10–14, >14), presence of defects in the vehicle (no, yes), presence of other passengers in the vehicle (no, yes);
- Environment variables: hour of the day (0–5, 6–11, 12–17, 18–23), area (urban or open road), type of road (highway or motorway, conventional road, street, other), intersection (no, yes), road use (peri urban area, ring road, residential, with special restrictions, other), traffic density (low, medium, high, very high), speed regulation (generic, specific); road surface (normal, altered), light conditions (daylight, twilight without artificial lighting, twilight with artificial lighting, darkness with artificial lightning, darkness without artificial lighting), meteorological conditions (normal, adverse);
- Crash severity (only minor injuries, major injuries, deathly victims). Major injuries were considered when the victim required > 24 h of hospitalization.

### 2.3. Analytic Strategy

The mediation analysis applied in the present study is based on the method proposed by Buis [26], a generalization of the original decomposition method developed by Erikson [27]. This method decomposes the total association between a categorical, discrete or continuous exposure, and an outcome in a direct effect and an indirect effect. As our outcome (*y*: whether or not a driver causes road crash) is binary, we used logistic regression to model it. According to Buis’ notation [26], let *x* be the age of the driver (for example, *x* = 1 are drivers aged 18–24, and *x* = 0 is the reference age group, i.e., 35–44), and *z* designs the set of environment- and vehicle-related mediators. According to our hypothesis (see Figure 1), the decomposition of the total effect of *x* upon *y* on a direct effect (*x* → *y***)** and an indirect effect (*x* → *z* → *y***)** can be estimated through the following Equation

(1) $$\frac{Ox=1,\;z|\;x=1}{Ox=0,\;z|\;x=0}=\;\frac{Ox=0,z|\;x=1}{Ox=0,\;z|\;x=0}\;\times \;\frac{Ox=1,\;z|\;x=1}{Ox=0,\;z|\;x=1}\;\mathrm{total}\;\;\;\;\;\;\;\;\;\;\;\;\;\;\;\;\;\;\;\;\;\;\;\;\;\;\;\;\mathrm{direct}\;\;\;\;\;\;\;\;\;\;\;\;\;\;\;\;\;\;\;\;\;\;\;\;\;\mathrm{indirect}$$

In Equation (1), ***O*** is the odds of *y* = 1 (causing a road crash). The first subscript represents the logistic regression coefficients and the second subscript represents the distribution of *z*. The left part of the equation (named as ‘total’) represents the OR that quantifies the global effect of *x* = 1 on *y*: the ***O*** of causing a road crash in drivers of the age group *x* = 1, divided by the ***O*** of causing a road crash in drivers of the age reference group (*x* = 0), given the observed values of *z* in each age group. The first term of the product (named ‘indirect’ in the equation) quantifies the indirect effect of *x* = 1 on *y*: the coefficients of the model are fixed so that they take values from the age reference category (*x* = 0), while *z* takes the observed values in each age category. Consequently, the numerator of this term is the ***O*** of *x* = 0 in the counterfactual situation in which *z* acquires the eigenvalue of *x* = 1. Therefore, this term expresses the OR for *x* = 1 that exclusively depends on the association between *x* and *z*. Finally, the second term of the product (named as ‘direct’ in the equation) refers to the OR of *x* = 1 on *y* which depends exclusively on its direct effect: the denominator is the ***O*** of *x* = 0 in the counterfactual situation in which *z* acquires the eigenvalue of *x* = 1. Therefore, the value of this OR exclusively depends on the direct association between *x* and *y*.

The model was implemented in Stata (version 15.0) (StataCorp^®^ 2019, College Station, TX, USA), with the ldecomp command. According to the theoretical framework explained above, the equations obtained from this command produced three OR estimates for each age group: an OR for the total effect of age; an OR for the DCP and an OR for the ICP (mediated through *z*). The original coefficients of the model shown in equation (1) were used to express the above decomposition in additive terms and thus determine the relative percentage contribution of each path to the total association.

First, a model was obtained for the entire sample including driver’s age, *z*, and also driver’s sex. In a second step, the model was obtained separately for men and women, and for crashes of low (only minor injuries), and high severity (resulting in major injuries or deaths).

To obtain the 95% confidence intervals of the OR estimations, Buis [26] proposes the use of the bootstrap method [28]. This is a procedure based on obtaining multiple samples from the population (with replacement), using the study sample as the population. It can estimate the standard error as the standard deviation of all point estimates obtained from the samples. Therefore, bootstrapping (1000 iterations) was used to obtain 95% confidence intervals for the estimated OR in each model.

## 3. Results

Table 1 shows the distribution of the 118,364 drivers included in the final sample of the study (the one which includes complete records for all variables, and for which the decomposition model was designed).

Table 2 shows, for the total sample and separately according to sex, the three OR values (total, DCP and ICP), and their corresponding 95% confidence intervals (CI) for all age groups of drivers, as well as the percentage contribution of DCP and ICP to the total OR.

Regarding the model obtained for the total sample, the risk of causing a crash was higher for the extreme-aged groups and reached its lowest value for drivers aged 35–44 years old. Compared to this age group, the highest risk was observed for the oldest drivers (>74 years old, total OR = 3.32). Most of this increase (92%) was linked to the DCP. Drivers aged 65–74 years old also showed an increased risk of crash (OR total = 1.65). In this group, ICP did not significantly contribute to this increased risk (OR = 1.00; 95% CI: 0.98–1.02). On the other hand, in the youngest age group (18–24 years old), the odds of causing a crash were 2.2 times higher than that of the 35–44 age group. Again, DCP contributed to the main part of this increase (89%).

The pattern described does not change substantially when stratifying the models by sex. In the group of younger drivers (<34 years), there are no differences between men and women in both the increased risk of causing a crash and the percentages of these increase associated with the DCP. For drivers over 54 years, the increased risk associated with older age is slightly higher in women (e.g., the total OR in over-74 group is 4.65 in women, and 3.13 in men). However, the percentage of these increases attributable to the DCP are slightly higher in men. In fact, men aged 65–74 showed a reverse sign weight of the component attributable to the ICP (Table 2).

Regarding the models stratified by severity of the crash shown in Table 3, we found no remarkable differences between both groups. In the younger drivers’ groups, there was a slightly higher increased risk of causing a crash, as well as the percentage contribution to the ICP, in the subgroup of more severe crashes. For drivers aged >45 years, the ICP contribution was lower for crashes resulting in major injuries or deaths. In fact, in this subgroup, the ICP yielded an OR lower than 1, (and, consequently, a negative percent contribution to the total OR) in the age groups from 55 to 74 years.

## 4. Discussion

First, our study confirms the relationship between the excess of risk of causing a crash and the extreme-aged groups of drivers (less than 24 and more than 74 years old), this risk being especially high for older drivers. Second, this excess risk in both groups is only partially explained by differences in the driving environment or in the vehicle driven. Therefore, we have to assume that age-related risky driving behaviors and loss of capabilities (which we have called DCP) are primarily responsible for these differences in both men and women.

We also found no substantial differences in this pattern when analyzing separately crashes with minor victims and crashes with major victims or deaths. In both groups, the DCP was also mainly responsible for the increased risks, and the ICP even showed a protective association with major-victim crashes in drivers aged 55–74.

We have not found previous studies based on a theoretical approach similar to ours. Therefore, direct comparisons of our results with previous ones are not possible. However, our results are consistent with those studies that show that drivers of extreme ages are involved in more crashes due to a riskier driving behavior rather than different environmental or vehicle circumstances. According to other studies, a driver’s error was the critical reason in 97% of crashes involving older drivers [14], and low-mileage bias has been reported to be insignificant in the rural context [29]. Regarding younger drivers, human factors were more influential than environmental factors in road crashes [1], especially executive function capacities and negative driving behavior [30]. In our media, it has been proposed that adolescents in higher academic grades and living in our region (Andalusia) were less aware of road safety [6].

All these studies pointed to intrinsic human behaviors and loss of capabilities as the main cause of traffic crashes in younger and older drivers. However, it has been proposed that low-mileage bias is an important factor overestimating older drivers’ risk in several studies [16,17]. These studies highlighted different environmental factors as the main reason for excess risk among older drivers, which are inconsistent with the results of our study. Our data did not deny the existence of this bias but showed that the main percentage of the risk was due to the DCP.

The analysis of all the riskier behaviors underlying this DCP, impossible to approach in a police-based database in our study, could be relevant not only to prevent future crashes, but also to better adapt to new automotive technologies safely, such as autonomous vehicles [31]. Behavioral studies may also help optimize preventive strategies in extreme-aged drivers in different contexts. This could potentially be decisive in reducing fatal crashes in developing countries, where fatalistic beliefs and risk-taking attitudes are key to road safety education [32]. In fact, as riskier behaviors are culturally determined and the age of drivers is also dependent on the distribution of the population pyramid, effective preventive strategies must be individualized for each country and context.

The DCP could also gather numerous mechanisms such as reckless behaviors, driving under the effects of alcohol, concentration disorder, delayed reactions, limited cognition, psychological loss of capabilities, dementia and other mental pathologies. Research aimed at quantifying those mechanisms in different subpopulations might also improve the individualization preventive strategies.

There is also an encouraging area of future research regarding different pathologies that may be associated with age, riskier behaviors and the risk of causing a road crash. In defining DCP, we mainly considered associated diseases and drug treatment when defining DCP. However, some middle-age diseases such as diabetes [33], cardiovascular disease or hypertension could have a considerable impact on driving abilities throughout life.

The results of our study suggest that human factors may explain the increased excess in risk of having a road crash in extreme-aged drivers, especially in the elderly. It seems essential to differentiate which part of the responsibility for a crash depends on a preventable misbehavior and which one on driving in an environment intrinsically associated with a higher crash risk. This difference has not been explored in depth in previous works and might make a difference in designing more precise preventive strategies.

Our study aimed at differentiating both components according to one of the main dependent human factors: age. It is important to note that our study does not attempt to identify which elements are intrinsically associated with a risker driving behavior in each age subgroup (it seems evident that those factors might be completely different in younger drivers—inexperience, alcohol abuse, etc.—than in older drivers—cognitive deterioration, pathologies, etc.). On the contrary, our study aims to identify which part of their respective excess risk of causing a road crash is not associated with this riskier driving behavior.

The practical implications of individualizing and quantifying both components of the association of age with the risk of causing a road crash could be widely exemplified. For instance, if (as our results suggest), the excess risk of causing a road crash in elderly drivers depends, predominantly, on a deterioration in their driving abilities (directly or indirectly related to aging), strategies focused on identifying these drivers with limitations in those skills and advise them to withdraw from traffic circulation might be appropriate. Interventions from Public Health institutions or Primary Health Care professionals focused on identifying potentially dangerous loss of driving abilities (ophthalmological evaluation, cognitive deterioration, prescribed drugs, etc.) and incorporating health advice on safety driving could be an excellent opportunity to address this issue. However, these strategies would not be effective if this excess risk depended on environmental circumstances alone (for example, driving in more hostile or unsafe driving environment or using damaged vehicles). In this hypothetical case, interventions could focus on informing these drivers and improving road safety in these environments.

Nevertheless, although the DCP prevails, environment-related prevention measures such as lower speed limits could well result in a substantial reduction in the frequency of serious accidents, in a possible interaction with individual cognitive impairments of older drivers.

This study has several limitations. Most of them are related to the data source: a police-based register with all the well-known drawbacks associated with this type of databases [34,35,36]: under-reporting of urban and less severe crashes, uncertainties about the validity of some variables, missing values for some of them, and lack of some other relevant variables to test our study hypothesis. For example, socio-economic factors and concrete risky driving behaviors could not be collected. Several studies attempted to develop a model for testing aberrant driving behaviors, such as the one tested by Zhang et al. [37], but in a police-based database it was impossible to collect some variables such as driver anger or hurry drivers. As an anonymous police-base register, we could not link the database to hospital records or clinical histories to enrich our data. We used a quasi-induced approach to design our control group. Although non-responsible drivers of clean collisions have been shown to constitute a representative sample of car drivers [22], a selection bias is still possible. On the other hand, we accepted the validity of our assumption about the allocation of responsibilities based on the commission of errors or infractions in clean collisions, which could be biased.

Future studies should focus on developing effective preventive strategies in extreme-aged drivers in order to decrease riskier driving behaviors.

## 5. Conclusions

In conclusion, our results support the hypothesis that most of the excess crash risk observed for the youngest and oldest drivers is primarily related to their higher frequency of risky driving behaviors or loss of capabilities and is much less dependent on the driving environment or on the vehicles they drove. This association was no different between men and women, or between crashes with minor or major victims. These results should be considered in order to prioritize preventive strategies intended to decrease road crashes among the youngest and oldest drivers. Future studies should be designed to focus on analyzing the concrete elements of these riskier driving behaviors, the identification and control of the potential loss of capabilities and exploring the usefulness of preventive programs for extreme-aged drivers.

## Figures and Tables

**Table 1:** Distribution of the study variables in the total sample of drivers and stratified by case and control groups.

Variable	Categories	Total Sample		Cases		Controls	
		N	%	N	%	N	%
Age	18–24	15,886	13.4	11,376	16.0	4510	9.6
	25–34	28,384	24.0	17,014	23.9	11,370	24.1
	35–44	29,194	24.7	15,735	22.1	13,459	28.5
	45–54	20,831	17.6	11,551	16.2	9280	19.7
	55–64	12,487	10.6	7238	10.2	5249	11.1
	65–74	7458	6.3	4965	7.0	2493	5.3
	>74	4124	3.5	3308	4.7	816	1.7
Sex	Male	78,387	66.2	48,558	68.2	29,829	63.2
	Female	39,977	33.8	22,629	31.8	17,348	36.8
Crash severity	Minor injuries	107,546	90.9	63,768	89.6	43,778	92.8
	Major injuries	8409	7.1	5750	8.1	2659	5.6
	Deaths	2409	2.0	1669	2.3	740	1.6
Zone	Open road	41,652	35.2	23,281	32.7	18,371	38.9
	Urban	76,712	64.8	47,906	67.3	28,806	61.1
Type of road	Highway-motorway	25,769	21.8	14,726	20.7	11,043	23.4
	Conventional road	48,970	41.4	31,650	44.5	17,320	36.7
	Street	38,024	32.1	21,182	29.8	16,842	35.7
	Other roads	5601	4.7	3629	5.1	1972	4.2
Road use	Peri urban	30,877	26.1	18,767	26.4	12,110	25.7
	Ring road	4398	3.7	2252	3.2	2146	4.6
	Residential	8350	7.1	4659	6.5	3691	7.8
	Special regulations	2827	2.4	1637	2.3	1190	2.5
	Other	71,912	60.8	43,872	61.6	28,040	59.4
Intersection	No	71,542	60.4	44,599	62.7	26,943	57.1
	Yes	46,822	39.6	26,588	37.4	20,234	42.9
Speed regulation	Generic	77,500	65.5	47,330	66.5	30,170	64.0
	Specific	40,864	34.5	23,857	33.5	17,007	36.1
Road Surface	Normal	100,224	84.7	58,764	82.6	41,460	87.9
	Altered	18,140	15.3	12,423	17.5	5715	12.1
Traffic density	Low	82,691	69.9	53,636	75.4	29,055	61.6
	Medium	21,003	17.8	11,528	16.2	9475	20.1
	High	12,817	10.8	5291	7.4	7526	16.0
	Very high	1853	1.6	732	1.0	1121	2.4
Hour of the day	0–5	6868	5.8	5444	7.7	1424	3.0
	6–11	30,981	26.2	18,915	26.6	12,066	25.6
	12–17	46,668	39.4	27,108	38.1	19,560	41.5
	18–23	33,847	28.6	19,720	27.7	14,127	29.9
Light conditions	Daylight	84,858	71.7	49,465	69.5	35,393	75.0
	Twilight, no artificial lights	4338	3.7	2781	3.9	1557	3.3
	Twilight, artificial lights	2841	2.4	1588	2.2	1253	2.7
	Darkness, artificial lights	13,307	11.2	7990	11.2	5317	11.3
	Darkness, no artificial lights	13,020	11.0	9363	13.2	3657	7.8
Weather	Good	96,388	81.4	56,754	79.7	39,634	84.0
conditions	Adverse	21,976	18.6	14,433	20.3	7543	16.0
Vehicle type	Car	103,520	87.5	61,725	86.7	41,795	88.6
	Van	11,335	9.6	7126	10.0	4209	8.9
	All-terrain	3509	3.0	2336	3.3	1173	2.5
Vehicle defects	No	116,720	98.6	69,743	98.0	46,977	99.6
	Yes	1644	1.4	1444	2.0	200	0.4
Years since the	0 to 4	19,548	16.5	10,554	14.8	8994	19.1
vehicle was	5 to 9	28,069	23.7	16,056	22.6	12,013	25.5
registered	10 to 14	37,849	32.0	23,012	32.3	14,837	31.5
	>14	32,898	27.8	21,565	30.3	11,333	24.0
Other passengers	No	76,866	64.9	49,853	70.0	27,013	57.3
	Yes	41,498	35.1	21,334	30.0	20,164	42.7
Total		118,364	100.00	71,187	60.1	47,177	39.9

**Table 2:** Total OR, Direct Causal Path OR and Indirect Causal Path OR to estimate the association between each age group of drivers and the odds of causing a road crash, in the total sample and stratified by sex.

(a) Total	Total Effect		Direct Causal Path			Indirect Causal Path		
Age Group	OR ^1^	95% CI ^2^	OR ^1^	95% CI ^2^	Percent Contribution to Total OR	OR ^1^	95% CI ^2^	Percent Contribution to Total OR
18–24	2.15	2.06–2.24	1.97	1.89–2.05	88.64	1.09	1.08–1.10	11.36
25–34	1.28	1.24–1.33	1.22	1.19–1.26	81.37	1.05	1.04–1.06	18.63
35–44	1	Reference	1	Reference		1	Reference	
45–54	1.06	1.02–1.10	1.04	1.00–1.07	61.91	1.02	1.01–1.03	38.09
55–64	1.16	1.11–1.21	1.12	1.08–1.17	78.24	1.03	1.02–1.05	21.76
65–74	1.65	1.57–1.75	1.66	1.57–1.75	100.50	1.00	0.99–1.02	−0.50
>74	3.32	3.07–3.60	3.02	2.78–3.27	91.94	1.10	1.08–1.12	8.06
**(b) Females**								
18–24	2.06	1.92–2.21	1.89	1.77–2.01	87.81	1.09	1.07–1.11	12.19
25–34	1.27	1.20–1.34	1.21	1.15–1.28	81.04	1.05	1.03–1.06	18.96
35–44	1	Reference	1	Reference		1	Reference	
45–54	1.14	1.08–1.21	1.10	1.04–1.17	73.57	1.04	1.02–1.05	26.43
55–64	1.46	1.35–1.58	1.35	1.25–1.46	78.80	1.08	1.06–1.11	21.20
65–74	2.31	2.04–2.61	2.12	1.88–2.40	89.84	1.09	1.06–1.12	10.16
>74	4.65	3.53–6.14	4.00	3.05–5.26	90.23	1.16	1.10–1.22	9.77
**(c) Males**								
18–24	2.19	2.08–2.31	2.02	1.92–2.13	89.84	1.08	1.07–1.10	10.16
25–34	1.30	1.24–1.35	1.24	1.19–1.29	82.66	1.05	1.03–1.06	17.34
35–44	1	Reference	1	Reference		1	Reference	
45–54	1.01	0.97–1.06	1.00	0.96–1.04	7.20	1.01	1.00–1.02	92.80
55–64	1.05	1.00–1.10	1.05	1.00–1.10	96.59	1.00	0.99–1.02	3.41
65–74	1.51	1.42–1.60	1.58	1.49–1.67	111.15	0.96	0.94–0.97	−11.15
>74	3.13	2.87–3.41	2.94	2.70–3.29	94.48	1.06	1.04–1.09	5.52

^1^ Odds Ratios (OR) were adjusted for sex of the driver in model (**a**). ^2^ 95% confidence intervals (CI) of each estimated OR.

**Table 3:** Total OR, Direct Causal Path OR and Indirect Causal Path OR to estimate the association between each age group of drivers and the odds of causing a road crash stratified by severity of the crash.

(a) Minor Victims	Total Effect		Direct Causal Path			Indirect Causal Path		
Age Group	OR ^1^	95% CI ^2^	OR ^1^	95% CI ^2^	Percent Contribution to Total OR	OR ^1^	95% CI ^2^	Percent Contribution to Total OR
18–24	2.10	2.01–2.19	1.94	1.87–2.02	89.48	1.08	1.07–1.10	10.52
25–34	1.28	1.23–1.32	1.22	1.18–1.26	81.78	1.05	1.04–1.06	18.22
35–44	1	Reference	1	Reference		1	Reference	
45–54	1.06	1.03–1.10	1.04	1.00–1.08	62.33	1.02	1.01–1.03	37.67
55–64	1.17	1.12–1.22	1.13	1.08–1.18	77.61	1.03	1.02–1.05	22.39
65–74	1.62	1.53–1.71	1.62	1.53–1.71	100.26	1.00	0.98–1.01	−0.26
>74	3.31	3.03–3.61	2.99	2.74–3.27	91.72	1.10	1.08–1.13	8.28
**(b) Major Victims and Deaths**								
18–24	2.68	2.30–3.13	2.25	1.93–2.63	82.23	1.19	1.14–1.24	17.77
25–34	1.37	1.21–1.56	1.27	1.12–1.43	74.05	1.09	1.05–1.21	25.95
35–44	1	Reference	1	Reference		1	Reference	
45–54	0.96	0.84–1.08	0.97	0.86–1.10	67.43	0.99	0.96–1.02	32.57
55–64	1.04	0.90–1.21	1.07	0.93–1.24	165.15	0.97	0.94–1.01	−65.15
65–74	1.75	1.47–2.08	1.88	1.58–2.24	113.05	0.93	0.89–0.97	−13.05
>74	2.94	2.31–3.74	2.89	2.27–3.68	98.40	1.02	0.96–1.08	1.60

^1^ Odds Ratios (OR) were adjusted for sex of the driver. Major victims are the ones that required hospitalization > 24 h. ^2^ 95% confidence intervals (CI) of each estimated OR.
